# Authors’ Response to Peer Reviews of “Finding Potential Adverse Events in the Unstructured Text of Electronic Health Care Records: Development of the Shakespeare Method”

**DOI:** 10.2196/31568

**Published:** 2021-08-11

**Authors:** Roselie A Bright, Summer K Rankin, Katherine Dowdy, Sergey V Blok, Susan J Bright, Lee Anne M Palmer

**Affiliations:** 1 US Food and Drug Administration Silver Spring, MD United States; 2 Booz Allen Hamilton McLean, VA United States; 3 US Food and Drug Administration Rockville, MD United States

*This is the author’s response to peer-review reports for the paper “Finding Potential Adverse Events in the Unstructured Text of Electronic Health Care Records: Development of the Shakespeare Method”*

This paper [1] first underwent review as two separate manuscripts: one on transfusion adverse events and the other on time-based adverse events.

In addition to responding to the reviewers’ comments [2-5], we made the following changes:

## Round 1 Review: Transfusion Adverse Events

### Anonymous [2]

#### General Comments

1. We believe our title matches the study contents. We do not understand how the results of using a new method, applied in a new area (blood transfusion adverse events [AEs]), are “self-evident.” We prefer to keep the title unchanged.
2. Please see the new subsection “Comparison of the Shakespeare Method to Other Applications of LDA Topic Modeling” at the end of the *Discussion* section:
  “We were unable to find published instances of LDA topic modeling applications for adverse event detection. Furthermore, we found none that apply LDA topic modeling to words or phrases in documents in the group of interest that are filtered to terms that most significantly distinguished a patient group of interest from a comparison group. This filtering process was essential for identifying topics describing the unique qualities of transfused vs nontransfused groups. Also, to our knowledge, we are the first to check the interpretation of documents with large numbers of topics with nontrivial scores.”
3. Please see the new subsection “Comparison of the Shakespeare Method to Other Applications of LDA Topic Modeling” at the end of the *Discussion* section for a summary of the use of latent Dirichlet allocation (LDA) topic modeling in electronic health record (EHR) data and how the Shakespeare method compares.
  We agree that natural language processing (NLP) is indispensable to finding potential AEs in unstructured text. Please see the new subsection “Comparison of the Shakespeare Method to Other Applications of LDA Topic Modeling” at the end of the *Discussion* section for the new text:
  “LDA topic modeling has been used for a variety of NLP tasks [6,7] (although it can also be used on other high-dimension data) such as text classification and filtering [8].”
  We state in the *Conclusions* section that the final step, manual interpretation of selected original notes, could benefit from adaptation of more sophisticated NLP methods.
4. As described, LDA topic modeling is one step in the Shakespeare method.
  In the *Discussion* section, “Comparison of the Shakespeare Method to Other Applications of LDA Topic Modeling subsection, we now say:
  “We were unable to find published instances of LDA topic modeling applications for adverse event detection. Furthermore, we found none that apply LDA topic modeling to words or phrases in documents in the group of interest that are filtered to terms that most significantly distinguished a patient group of interest from a comparison group. This filtering process was essential for identifying topics describing the unique qualities of transfused vs nontransfused groups. Also, to our knowledge, we are the first to check the interpretation of documents with large numbers of topics with nontrivial scores.”
5. Thank you for pointing out this error. We have made the correction to five steps.
6. We have clarified this sentence in the *Introduction* section, “EHRs for Postmarketing Surveillance” subsection, and made a similar change to the *Background* section in the abstract. The new paragraph is:
  “Many methods for finding AEs in text [9-34] rely on predefining possible AEs before searching for prespecified words and phrases or manual labeling (standardization) by investigators. Crucially, events described in text may not necessarily be attributed to AEs [19,35,36]. We wanted to develop a method to identify possible AEs, even if unknown or unattributed, without any prespecifications or standardization of notes.”

### Anonymous [3]

#### General Comments

We have clarified our statements in the *Introduction* section, “Selection of Case of Blood Transfusion” subsection, to indicate that some transfusion AEs were established in the literature by 2002 while others were gaining recognition over the time of the data set (2001-2012).

#### Specific Comments

##### Major Comments

1. We are in the process of publishing the code and expect to have a permanent citation in a few weeks. We now cite it as reference 54 in the *Methods* section, “The Shakespeare method” subsection.
2. The details are in another paper we cited (reference 57).
3. We added some explanation to the *Methods* section, “Step 4. Model Topics” subsection:
  1. “An important consideration for LDA is that the number of topics must be selected a priori. The results of topic modeling change depending on the number of topics assigned to a corpus—this is an iterative (hyperparameter tuning) process that requires human judgment to interpret the topics (based on the top terms in each topic) and determine which number of topics best fits the corpus. With too few topics assigned, topics are not cohesive and do not add any clarity or information to an analysis. With too many topics assigned, “incoherent” topics that do not capture terms common to the member documents proliferate; also, useful topics are likely split among smaller, more specific topics, although that does not limit the ability to analyze true clusters in the corpus.
    To tune the hyperparameters of the LDA model, we calculated models with the following numbers of topics: 25, 35, 45, 55, 65, 75, 85. We observed (data not shown):
    In the *Discussion* section, “Comparison of the Shakespeare Method to Other Applications of LDA Topic Modeling” subsection, we added:
    “The chosen number of topics was effective for identifying a range of PTAEs. Evaluation of the overlap of topics and contents of documents identified for varying numbers of topics has not been reported in the literature. Our iterative approach to evaluating different hyperparameters demonstrated to our satisfaction the relative stability of PTAEs indicated by topics.
    We determined the number of topics based on our experience of tuning the hyperparameters, the number of TAEs reported in the literature, and the complexities of critical care patients. We were satisfied with the number because there was both overlap of topics that simultaneously had high word and document scores and some incoherent topics with low scores. As the number of topics gets too large, additional topics are uninterpretable, and that as data set size increases, more robust topics are generated [37].”
  2. In the *Discussion* section, “Comparison of the Shakespeare Method to Other Applications of LDA Topic Modeling” subsection, we added:
    “Systematic evaluation of the number of topics and other hyperparameters is always necessary for LDA topic modeling in a new setting.”
  3. In the *Methods* section, “Step 4. Model Topics” subsection, we added:
    “Topic modeling is an unsupervised method commonly used in NLP to extract the most relevant terms for each topic (cluster) of similar documents [6,7]. We chose latent Dirichlet allocation (LDA) [8] to accomplish topic modeling of the T documents. LDA is a generative probabilistic model that results in interpretable dimensionality reduction, which means that we reduced 41,664 terms to 45 topics for our data. A topic is a multimodal distribution of terms over an entire vocabulary (in our case, all the filtered terms). A topic consists of co-occurring terms in this corpus of T documents. Each document can have a mixture of these topics. Each topic contribution in a document is a probability (we refer to this as a document topic score); thus, the scores of all topics for a document sum to 1 (see Figure 3D).”
4. In the new *Discussion* section, “Use of Classification to Filter Document Vectors” subsection, we added:
  “As noted before, we were initially surprised that primarily unigrams (and not the longer sequences) appeared to play a significant role in distinguishing transfusion from control texts. We believe it is possible that enough unigrams that were part of meaningful phrases were also in other phrases or were significant on their own to result in relatively higher scores. For example, although “mechanical ventilation” conveys more meaning than just “mechanical” or “ventilation,” each word occurs singly or in phrases other than “mechanical ventilation.” Because bigrams and phrases were important in other LDA studies [38,39], we do not conclude that our unigram finding is necessarily applicable to other study settings. In this data set and blood transfusion situation, including only unigrams would not be expected to have changed the particular unigrams selected during the ensemble classification step. In other studies, it might be important to include n-grams where n>1.”
  In the new *Discussion* section, “Use of Classification to Trim Document Vectors” subsection, we added:
  “In this data set and blood transfusion situation, including only unigrams would not be expected to have changed the particular unigrams selected during the ensemble classification step. In other studies, it might be important to include n-grams where n>1.”
  In the new *Discussion* section, “Use of Classification to Trim Document Vectors” subsection, we added:
  “Because bigrams and phrases were important in other LDA studies [38,39], we do not conclude that our unigram finding is necessarily applicable to other study settings.”
5. We agree. In the *Conclusion* section, we added:
  “We present our use of the Shakespeare method for a different surveillance question elsewhere [40].”
6. The renamed *Methods* subsection “Step 3. Extract Significant Terms” now explains the filtering (trimming) method in more detail.
  In the new *Discussion* section, “Use of Classification to Filter Document Vectors” subsection, we added:
  “Filtering the vectors to only terms that were important for focusing the topics on clinical conditions specific to transfusion, including reasons for and consequences of transfusion, was important for identifying PTAEs.”

##### Minor Comments

1. We simplified the statement to:
  “We chose the case of transfusion adverse events (TAEs) and potential TAEs (PTAEs) because new TAE types were becoming recognized during the study data period, so we anticipated an opportunity to find unattributed TAEs in the notes.”
2. Thank you for finding this mistake, which we corrected to “five steps.”
3. Thank you for finding this typo in the *Conclusion* section. “Her” should have been “EHRs” and has been corrected.

## Round 2 Review: Transfusion Adverse Events

We finalized the citation for the Shakespeare method software in reference 54, and submitted manuscripts with and without tracked changes that show our changes.

We believe we addressed the reviewer’s [2] concerns. We apparently did not because some of the prior concerns remain in this review round. We are puzzled by the newly restated comments and would like more clarity on his/her points so that we can be sure to address the concerns. We provide more details about our questions as individual responses below.

### Anonymous [2]

#### General Comments

We disagree that the Shakespeare method is an alternative to NLP, because we leverage NLP, which includes many methods. As part of the Shakespeare method, we used the following NLP methods: n-gram formation, count vectorization, supervised learning, and LDA topic modeling. We mentioned another NLP method, word/phrase searches, in the *Introduction* section, thus demonstrating our understanding of that method; we also discussed why we did not choose to use it. To form the transfused and nontransfused groups, we created and used a dictionary of transfusion terms. Outside of our paper, we are, indeed, familiar with many other NLP methods (stemming, sentence boundary recognition, part-of-speech tagging, parsing, semantics, sentiment analysis, word sense disambiguation, language models, language translation, and neural network–based machine learning) that are a menu of methods that may or may not be useful for a particular application. We do not understand why the reviewer thinks we do not understand NLP, why the reviewer thinks NLP is the preferred alternative to the Shakespeare method, and why that means we might be making mistaken conclusions.

#### Specific Comments

##### Major Comments

1. The reviewer seems to agree that the dictionary method relies on predefined possible AEs, which could rely on, for example, the Unified Medical Language System vocabulary list and could miss important terms. We are proposing an alternative method to find both expected and unexpected possible AEs, as we state in the *Introduction* section. We do not understand what the criticism is.
2. We agree and state in the *Discussion* section that in addition to possibly causal TAEs, the Shakespeare method identified reasons for transfusion, consequences of reasons for transfusion, and possibly noncausal PTAEs. We agree and state that the PTAEs need manual review to distinguish among these groups. As we state, the difference from the NLP dictionary method is that the Shakespeare method found PTAEs that were not described as related to transfusion in the notes or billing codes. The dictionary method cannot find potentially important terms and phrases that are not in the dictionary.
3. The application of the Shakespeare method to blood transfusion is a use scenario, so we do not understand why the reviewer thinks a potential use scenario needs to be included; however, we did include reference 107 as an additional scenario. We do not understand why or how manual review is an example of a potential use scenario. We reported our manual review of the results, so we do not understand what the reviewer means by asking “will more manual reviews be needed for the results.”

## Round 1 Review: Time-Based Adverse Events

### Reviewer CD

#### General Comments

1. We changed the beginning of the sentence to “We examined whether.”
2. Thank you!
3. We already stated some limitations. In the subsection “Discussion of Time Periods Case,” we pointed out that removing numerals from alphanumeric words had resulted in the creation of a “junk” topic that we would not recommend doing again. Additionally, in the *Conclusions* section, we mentioned that further development of tools for evaluating the reports would be very helpful. Furthermore, in the subsection “Use of Classification to Filter Document Vectors,” we added our observation that only unigrams survived the classification process in both the transfusion and time periods cases, and declined to recommend only using unigrams in other settings.

### Reviewer CI

#### General Comments

We appreciate the reviewer’s praise and hope we have satisfied the concerns.

#### Specific Comments

##### Major Comments

1. We agree that it would be great to know the accuracy of the Shakespeare method. Please see “Top-Scoring Documents for Each Transfusion Topic,” where we reviewed a random selection of transfusion admissions and compared them to the transfusion documents with high topic scores.

##### Minor Comments

1. We trimmed the list of keywords.
2. We are satisfied with the current state of the *Conclusions* section.

## Abbreviations

AE: adverse event
EHR: electronic health record
LDA: latent Dirichlet allocation
NLP: natural language processing
